# Lack of NPR1 Increases Vascular Endothelial Adhesion through Induction of Integrin Beta 4

**DOI:** 10.3390/ijms232012627

**Published:** 2022-10-20

**Authors:** Hongfei Liu, Jiankun Liu, Changkun Long, Liping Chen, Wenxing Zhan, Wanli Xiao, Xueting Gong, Man Liu, Xiao-Li Tian, Shenghan Chen

**Affiliations:** 1 Vascular Function Laboratory, Human Aging Research Institute, School of Life Science, Jiangxi Key Laboratory of Human Aging, Nanchang University, Nanchang 330031, China; 2 Aging and Vascular Diseases, Human Aging Research Institute, School of Life Science, Jiangxi Key Laboratory of Human Aging, Nanchang University, Nanchang 330031, China; 3 Metabolic Control and Aging, Human Aging Research Institute, School of Life Science, Jiangxi Key Laboratory of Human Aging, Nanchang University, Nanchang 330031, China

**Keywords:** cell adhesion, endothelial cells, integrin beta 4, NPR1

## Abstract

Natriuretic peptide receptor 1 (NPR1) serves as a modulator of vascular endothelial homeostasis. Interactions between monocytes and endothelial cells may initiate endothelium dysfunction, which is known as an early hallmark of atherosclerosis. In this study, we performed RNA-sequencing analysis for the aorta of *Npr1* knockout (*Npr1^+/−^*) mice and found that differentially expressed genes were significantly related to cell adhesion. This result was supported by an increased expression of intercellular adhesion molecule 1 (ICAM-1) in the aortic endothelium of *Npr1^+/−^* mice. Moreover, we observed that the knockdown of *NPR1* increased ICAM-1 expression and promoted THP-1 monocyte adhesion to human umbilical vein endothelial cells (HUVECs). *NPR1* overexpression decreased ICAM-1 expression and inhibited the adhesion of monocytes to HUVECs treated by TNF-α (a cell adhesion inducer). Further analysis showed that adhesion-related genes were enriched in the focal adhesion signaling pathway, in which integrin beta 4 (*Itgb4*) was determined as a key gene. Notably, ITGB4 expression increased in vascular endothelium of *Npr1^+/−^* mice and in *NPR1*-knockdown HUVECs. The deficiency of ITGB4 decreased ICAM-1 expression and attenuated monocyte adhesion to *NPR1*-knockdown endothelial cells. Additionally, a reduced NPR1 and an increased ITGB4 expression level were found in an atherosclerosis mouse model. In conclusion, our findings demonstrate that NPR1 deficiency increases vascular endothelial cell adhesion by stimulating ITGB4 expression, which may contribute to the development of atherosclerosis.

## 1. Introduction

Vascular endothelium is a single layer of endothelial cell lining of blood vessels, which plays a critical role in maintaining vascular homeostasis [1,2]. Impairment of the endothelium leads to an increase in leukocyte adhesion, endothelial permeability, and inflammatory cytokines, in which the adhesion of monocytes to the endothelium is recognized as an early stage of atherosclerosis [3,4,5]. Atherosclerosis is associated with myocardial infarction, coronary artery disease, stroke, and peripheral vascular disease [6]. Genetic elements are of importance for the elevated risk of atherosclerosis and many genes have been implicated in the initiation and progression of atherosclerosis [7,8], including integrin beta 4 (*ITGB4*) [9], intercellular adhesion molecule-1 (*ICAM-1*) [10], and natriuretic peptide receptor 1 (*NPR1*) [11]. ITGB4 is one of the cell surface laminin receptors that is detected in vascular endothelial cells [12,13]. ITGB4 is indispensable for adhesion, proliferation, apoptosis, and senescence, which are important factors in the development of atherosclerosis [9]. An elevated ITGB4 level has been found in mice with atherosclerosis [14]. Likewise, upregulation of ICAM-1 is a feature of endothelial dysfunction [15]. ICAM-1 expression increased with endothelial cell activation, which promotes the recruitment and adhesion of circulating leukocytes to the vessel wall [16]. ICAM-1 is also considered as a marker of pre-clinical atherosclerosis [10]. Increased ICAM-1 expression in vascular endothelial cell promotes leukocyte–endothelial cell adhesion [17,18,19]. Moreover, atrial natriuretic peptide (ANP) reduces the expression of ICAM-1 [20]. NPR1 is a transmembrane receptor that is activated by ANP or B-type natriuretic peptide to synthesize cyclic guanosine monophosphate (cGMP) [21,22]. A lack of NPR1 results in high blood pressure and hypertensive heart disease [21]. Importantly, hypertension is associated with endothelial dysfunction, which increases the susceptibility to atherosclerosis [23]. A study shows that ANP-NPR1-cGMP cascade may be involved in the prevention of increased arterial endothelial cell permeability and atherogenesis [24]. *Npr1*-deficient mice on an apolipoprotein E (*Apoe*) knockout background exhibit more severe atherosclerotic lesion compared with mice with *Apoe*-knockout only [11]. To date, the underlying mechanism of NPR1 on the process of atherosclerosis remains undetermined. 

Very recently, we reported that NPR1 is essential to regulate endothelial cell senescence and vascular aging [25]. Subsequently, we performed RNA-sequencing analysis for the aorta of *Npr1* knockout (*Npr1^+/−^*) mice to search for novel genes associated with vascular aging. Interestingly, we found that the differentially expressed genes (DEGs) were significantly enriched in the signal pathway related to cell adhesion. Therefore, in this study, we investigated the effect of NPR1 on cell adhesion at the cellular level and in the different mouse models. We demonstrate that NPR1 participates in modulating monocyte–endothelial cell adhesion.

## 2. Results

### 2.1. NPR1 Deficiency Increases Vascular Endothelial Cell Adhesion

**Figure 1:**
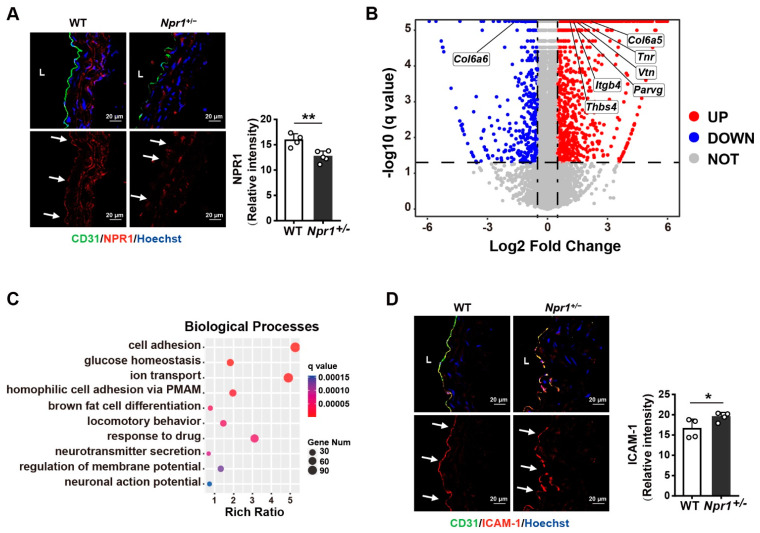
Increased vascular endothelial cell adhesion by *NPR1* deficiency. (**A**) Immunofluorescent staining for NPR1 (red), endothelium (green), and nuclei (blue) in aorta frozen sections from *Npr1*^+/-^ and WT mice. *n* = 4. Scale bar: 20 µm. L: Lumen. Arrows indicate the endothelium. Relative expression levels were calculated according to mean fluorescent intensity. (**B**) Volcano plots of differentially expressed genes in aorta of *Npr1*^+/-^ and WT mice. *n* = 3. |log2 fold change| ≥ 0.5, q value < 0.05. *Col6a6*: collagen type VI alpha 6 chain; *Thbs4*: thrombospondin 4; *Itgb4*: integrin beta 4; *Col6a5*: collagen type VI alpha 5 chain; *Tnr*: tenascin R; *Vtn*: vitronectin; *Parvg*: parvin gamma. (**C**) GO annotation of differentially expressed genes in the aorta of *Npr1*^+/-^ and WT mice. *n* = 3. PMAM: plasma membrane adhesion molecules. Y-axis indicates biological processes and X-axis indicates the rich ratio. The intensity of color and bubble size denote the q value and gene number, respectively. (**D**) Immunofluorescent staining for ICAM-1 (red), endothelium (green), and nuclei (blue) in the aorta of *Npr1*^+/-^ and WT mice. *n* = 4. Scale bar: 20 µm. L: Lumen. Arrows indicate the endothelium. Relative expression levels were calculated according to the mean fluorescent intensity. Data are mean ± S.D. * *p* < 0.05, ** *p* < 0.01.

In *Npr1^+/−^* mice, a decreased NPR1 expression in the aorta, especially in the endothelium, was confirmed by immunofluorescent staining (Figure 1A). We next performed RNA-sequencing analysis for the aorta of *Npr1^+/−^* and wild type (WT) mice. A total of 2184 DEGs (|log2 fold change| ≥ 0.5, q value < 0.05) was found (Figure 1B). Gene Ontology (GO) biological process annotation showed that these DEGs were highly related to cell adhesion among the top 10 signaling pathways (Figure 1C). Supportively, immunofluorescent staining showed an increased expression of ICAM-1 (an adhesion marker) in the aortic endothelium of *Npr1^+/−^* mice compared with that of WT (Figure 1D), suggesting that NPR1 may be associated with endothelial cell adhesion. 

### 2.2. Endothelial Cell Adhesion Is Enhanced by Knockdown of NPR1 but Reduced by Overexpression of NPR1

**Figure 2:**
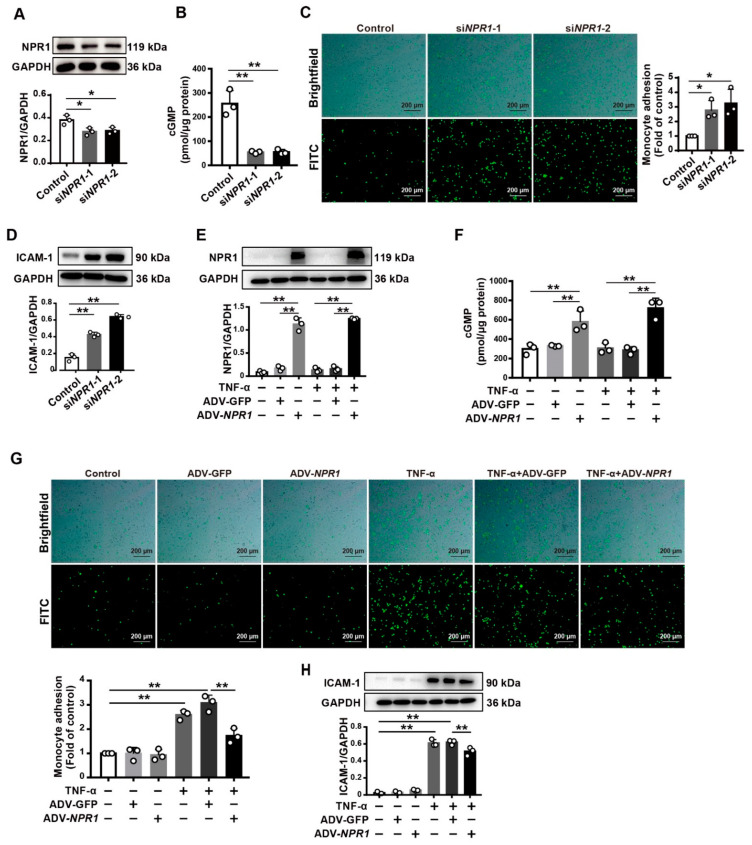
Altered adhesion of monocytes to HUVECs by knockdown or overexpression of *NPR1*. (**A**) Western blotting for NPR1 expression in HUVECs transfected with siRNAs (si*NPR1*-1 and si*NPR1*-2). Quantitative data were analyzed by densitometry. (**B**) Levels of cGMP in HUVECs transfected with siRNAs (si*NPR1*-1 and si*NPR1*-2). (**C**) Monocyte (green)–endothelial cell adhesion assay for HUVECs transfected with si*NPR1*-1 and si*NPR1*-2. Scale bar: 200 µm. Relative adhesion was evaluated by monocyte counts. (**D**) Western blotting for ICAM-1 expression in HUVECs transfected with siRNAs (si*NPR1*-1 and si*NPR1*-2). Quantitative data were analyzed by densitometry. (**E**) Overexpression of *NPR1* in HUVECs treated with TNF-α or GFP as a control. (**F**) Levels of cGMP in HUVECs transfected with overexpression *NPR1*. (**G**) Monocyte (green)–endothelial cell adhesion assay for TNF-α-treated HUVECs with overexpressed *NPR1*. Relative adhesion was evaluated by monocyte counts. Scale bar: 200 µm. (**H**) Western blotting for ICAM-1 expression in HUVECs transfected with overexpression of *NPR1*. Quantitative data were analyzed by densitometry. Data are mean ± S.D. * *p* < 0.05, ** *p* < 0.01.

To test our hypothesis, we carried out monocyte–endothelial cell adhesion assay in *NPR1*-knockdown or -overexpressed human umbilical vein endothelial cells (HUVECs). Western blot analysis showed a reduction in NPR1 protein levels in HUVECs transfected with two sets of siRNAs (si*NPR1*-1 and si*NPR1*-2), compared with the non-targeting control siRNA (Figure 2A). The level of cGMP was decreased in *NPR1*-knockdown HUVECs (Figure 2B). Subsequently, we observed that the adhesion of monocytes (THP-1) to HUVEC was enhanced in the cells with *NPR1* knockdown, without differences of endothelial cell density on brightfield and FITC microscopy (Figure 2C), indicating that increased monocyte adhesion by the knockdown of *NPR1* is not due to an increase in cell number. Additionally, the knockdown of *NPR1* enhanced the expression of ICAM-1 in HUVECs (Figure 2D). Moreover, the overexpression of *NPR1* (Figure 2E) increased the cGMP level (Figure 2F) and inhibited monocyte adhesion in HUVECs with treatment of TNF-α, a cell adhesion inducer [26] (Figure 2G). ICAM-1 expression was inhibited by overexpression of *NPR1* in HUVECs treated with TNF-α (Figure 2H). All of these results indicate that NPR1 is involved in modulating vascular endothelial cell adhesion.

### 2.3. Integrin Beta 4 (Itgb4) Is Identified as a Key Gene Related to Focal Adhesion under NPR1 Deficiency 

**Figure 3:**
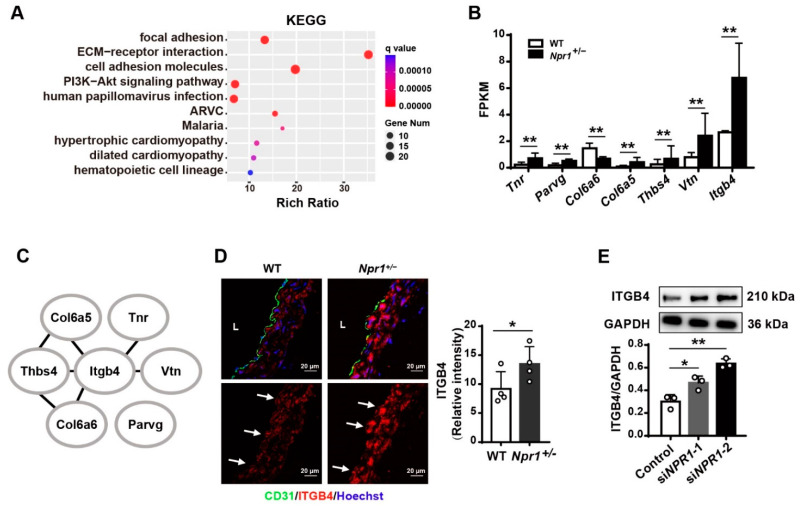
Identification of *Itgb4* in the aorta of *Npr1*^+/-^ mice and in *NPR1*-knockdown HUVECs. (**A**) KEGG enrichment of genes related to cell adhesion. ARVC: arrhythmogenic right ventricular cardiomyopathy. Y-axis represents pathways and X-axis represents the rich ratio. The intensity of color and bubble size indicate the q value and gene number, respectively. (**B**) mRNA expression of focal adhesion-related genes in the aorta of *Npr1*^+/-^ and WT mice. (**C**) A gene interaction network for targeted genes related to focal adhesion in the aorta of *Npr1*^+/-^ mice. (**D**) Immunofluorescent staining for ITGB4 (red), endothelium (green), and nuclei (blue) in the aorta of *Npr1*^+/-^ and WT mice. *n* = 4. Scale bar: 20 µm. L: Lumen. Arrows indicate the endothelium. Relative ITGB4 levels were evaluated based on the mean fluorescent intensity. (**E**) Western blot analysis for ITGB4 expression in *NPR1*-knockdown HUVECs. Data are mean ± S.D. * *p* < 0.05, ** *p* < 0.01 vs. WT or control.

To address how NPR1 regulates vascular endothelial adhesion, we further performed Kyoto Encyclopedia of Genes and Genomes (KEGG) analysis on cell-adhesion-related genes. We found that these genes were enriched in pathways linked to focal adhesion, ECM–receptor interaction, cell adhesion molecules, PI3K-Akt signaling pathway, and cardiomyopathy. Among them, focal adhesion was the one with most significant change (Figure 3A), in which seven genes were screened out (|log2 fold change| ≥ 1, q value < 0.05) (Figure 1B and Figure 3B). Gene interaction network analysis identified *Itgb4* as a key node gene in the signaling network (Figure 3C). Through immunofluorescent staining, we found that ITGB4 expression level was higher in the aorta, especially in the endothelium, of *Npr1^+/−^* mice than that of WT mice (Figure 3D). Through Western analysis, it was observed that ITGB4 expression was increased in *NPR1*-kncokdown HUVECs (Figure 3E). Furthermore, ITGB4 binds to HLA class I (HLAI) as a complex that strengthens monocyte adhesion via ICAM-1 clustering on endothelial cells by the mTOR signaling pathway [27]. All of the data suggest that NPR1 deficiency promotes the ITGB4 expression level. 

### 2.4. NPR1 Suppresses Monocyte–Endothelial Cell Adhesion by Reducing ITGB4 Expression

**Figure 4:**
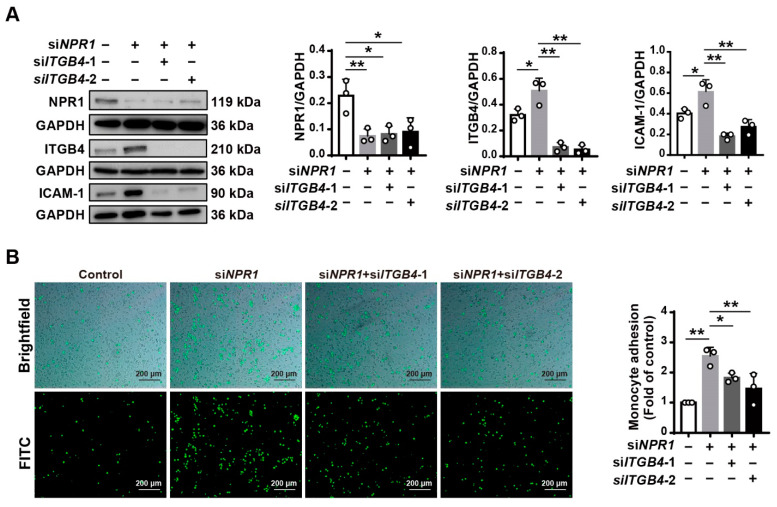
Decreased cell adhesion induced by NPR1 inhibition in *ITGB4*-knockdown HUVECs. (**A**) Western blot analysis for NPR1, ITGB4, and ICAM-1 expression in HUVECs treated with si*NPR1* and followed by transfection with si*ITGB4*-1 and si*ITGB4*-2. Analysis of endogenous NPR1 and ITGB4 levels, normalized to GAPDH, by densitometry, respectively. (**B**) Monocyte (green)–endothelial adhesion assay of HUVEC treated with si*NPR1* and followed by transfection with si*ITGB4*-1 and si*ITGB4*-2. Scale bar: 200 µm. Data are mean ± S.D. * *p* < 0.05, ** *p* < 0.01.

To verify the above results, we simultaneously knocked down *NPR1* and *ITGB4* in HUVECs. HUVECs were co-transfected with si*NPR1* and si*ITGB4* and then incubated for 48 h. Western analysis showed that the expression levels of NPR1 and ITGB4 were significantly diminished, indicating a high knockdown efficiency (Figure 4A). In these cells, we found that monocyte adhesion to HUVECs was evidently reduced in the presence of si*ITGB4*-1 and si*ITGB4*-2 compared with those exposed to si*NPR1* only, and no differences in endothelial cell density were observed on brightfield and FITC microscopy (Figure 4B), suggesting that decreased monocyte adhesion by the knockdown of *ITGB4* in *NPR1*-deficient cells is not due to a loss of endothelial cells. Moreover, the knockdown of *ITGB4* decreased the expression of ICAM-1 in HUVECs treated with si*NPR1* (Figure 4A). The results demonstrate that NPR1 decreases endothelial cell adhesion by inhibiting ITGB4 and ICAM-1 expression.

### 2.5. Atherosclerosis Mouse Model Exhibits Decreased NPR1 and Increased ITGB4 Expression 

**Figure 5:**
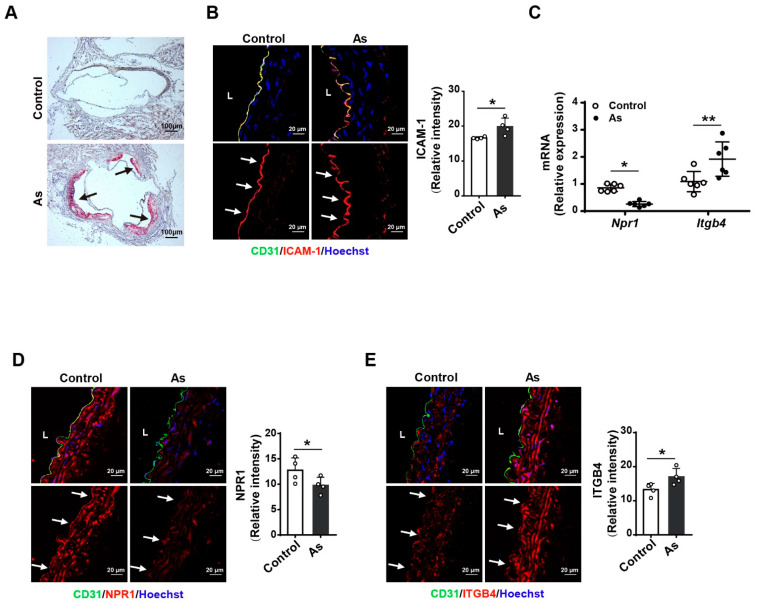
NPR1 and ITGB4 expression in the aorta from a mouse model of atherosclerosis (As). (**A**) Oil red O staining of mouse aortic root in the As model. Scale bar: 100 µm. (**B**) Immunofluorescent staining for ICAM-1 (red), endothelium (green), and nuclei (blue) in aorta frozen sections from the mouse model of As. *n* = 4. Scale bar: 20 µm. L: Lumen. Arrows indicate the endothelium. Relative expression levels were calculated according to the mean fluorescent intensity. (**C**) qPCR for *Npr1* and *Itgb4* mRNA expression in mouse aorta from the As model. (**D**, **E**) Immunofluorescent staining for NPR1 (red), ITGB4 (red), endothelium (green), and nuclei (blue) in aorta frozen sections from the mouse model of As. *n* = 4. Scale bar: 20 µm. L: Lumen. Arrows indicate the endothelium. Relative expression levels were calculated according to the mean fluorescent intensity. Data are mean ± S.D. * *p* < 0.05, ** *p* < 0.01 vs. WT.

It is known that an increase in monocyte adhesion to the endothelium is an underlying mechanism of atherosclerosis [28]. Thus, we further examined the expression of NPR1 and ITGB4 in the aorta from a mouse model of atherosclerosis. Oil red O staining showed a marked plaque in the aortic root (Figure 5A). Higher ICAM-1 expression was detected in the aortic endothelium from this model (Figure 5B). At mRNA levels, *Npr1* expression was decreased and *Itgb4* expression was increased in the aorta of atherosclerotic mice compared with that of the control mice (Figure 5B). Moreover, the protein expression levels of NPR1 in reduction (Figure 5D) and ITBG4 in augmentation (Figure 5E) were observed in the aortic endothelium of these mice. All of the data suggest that NPR1-ITGB4 may participate in atherosclerosis development. 

## 3. Discussion

Our study shows that *Npr1^+/−^* mice present an increased cell adhesion in the aortic endothelium. The knockdown of *NPR1* in HUVECs promotes monocyte adhesion to endothelial cells, while the overexpression of *NPR1* inhibits monocyte–endothelial cell interaction. Moreover, NPR1 deficiency increases ITGB4 expression in the endothelial cells and endothelium of *Npr1^+/−^* mice. At the cellular level, knockdown of *ITGB4* decreases the adhesion of monocytes to *NPR1*-deficient endothelial cells. Furthermore, the atherosclerosis mouse model displays a decreased NPR1 and an increased ITGB4 expression. These results suggest that NPR1-ITGB4 signaling may modulate endothelial cell adhesion, probably impacting the processes of atherosclerosis. 

NPR1, as the receptor of natriuretic peptides, is responsible for controlling blood pressure and is also involved in inflammatory response [29,30,31,32]. High blood pressure may cause endothelial dysfunction that triggers proinflammatory cytokines, which promotes adhesion molecule expression, thereby inducing vascular inflammation and atherogenesis [33,34]. Our recent study shows that NPR1 deficiency causes endothelial dysfunction marked by augmented interleukin 6 and interleukin 8, elevated reactive oxygen species (ROS) production, and decreased endothelial nitric oxide synthase (eNOS) and nitric oxide (NO) levels in vitro and in vivo [25]. Accumulating evidence indicates that endothelial dysfunction is the feature of atherosclerosis and the interaction of monocytes and endothelial cells is an initiated step of endothelium dysfunction [3]. Adhesion molecules such as ICAM-1 may enable monocyte adhesion to endothelial cells [35]. In the inflamed endothelium, the expression level of ICAM-1 is elevated, which facilitates the formation of atherosclerosis [15,36]. In *Npr1^+/^^−^* mice, we observed an increased ICAM-1 expression in the aortic endothelium. Studies have revealed that ICAM-1 plays a central role in regulating monocyte recruitment to vascular endothelium [37,38]. Monocyte recruitment is pivotal for developing plaques in atherosclerotic mice [39,40,41]. Our data show that the knockdown of NPR1 stimulates monocyte adhesion to vascular endothelial cells, whereas the overexpression of *NPR1* attenuates monocyte–endothelial cell interaction induced by TNF-α. These findings support an essential role of NPR1 in modulating endothelial cell adhesion with monocytes.

Based on RNA-sequencing analysis of the aorta of *Npr1^+/−^* mice, we found that DEGs related to cell adhesion were mainly enriched in the focal adhesion signaling pathway. Among seven genes identified in this pathway, ITGB4 is considered to be a key gene in the signaling network. ITGB4 is a transmembrane receptor and is primarily expressed on the cell membrane of different cell types including endothelial cells, which mediates cell to cell adhesion, migration, and apoptosis [13,42,43,44]. ITGB4 also impacts vascular remodeling by TGF-β cascade [13]. The interaction of ITGB4 cytoplasmic domain and elements at the specific structures facilitates endothelial cell adhesion [45]. Moreover, cell adhesion interaction is governed by N-glycosylation as well as phosphorylation of ITGB4 [46,47]. A mouse model of ITGB4 with deletion of cytoplasmic signaling domain reduces the endothelial inflammatory response induced by mechanical stress [48], while mice with atherosclerotic plaques display an increased ITGB4 [9]. Recently, a study found that the HLA I/ITGB4 complex stabilizes monocyte adhesion via ICAM-1 aggregation on endothelial cells in an mTOR-dependent manner [27]. This evidence indicates that ITGB4 participates in endothelial cell adhesion, inflammation, and atherogenesis. We demonstrate that ITGB4 and ICAM-1 expression is augmented in the aortic endothelium of *Npr1^+/−^* mice and in endothelial cells with *NPR1*-knockdown. Notably, the lack of ITGB4 reduces ICAM-1 expression and prevents monocyte adhesion to *NPR1*-knockdown endothelial cells. These results reveal that the effect of NPR1 on monocyte attachment to endothelial cells may be achieved by controlling ITGB4 expression. 

A previous study has reported that *Npr1* deficiency increases the atherosclerotic plaque area in *Apoe*-knockout mice, indicating that NPR1 exacerbates the progress of atherosclerosis [11]. We also found that NPR1 mRNA and protein expression were decreased in the endothelium of atherosclerotic mice, while ITGB4 expression was elevated. Therefore, it is possible that NPR1-ITGB4 contributes to atherosclerosis by governing monocyte adhesion to the vascular endothelium. In addition, ANP has been found to promote to innate immunity by suppressing the release of proinflammatory factors and inhibiting the expression of adhesion molecules [20,49]. Hence, the ANP–NPR1–ITGB4 signaling axis may became a molecular target in a therapeutic strategy for atherosclerosis. 

## 4. Material and Methods

### 4.1. Cell Culture

HUVECs were freshly isolated from the umbilical cord and identified by endothelial cell markers. HUVECs were maintained in Endothelial Cell Medium (ScienCell, San Diego, CA, USA). Human monocytic cell line (THP-1) (Procell, Wuhan, Hubei, China) was cultured in RPMI-1640 medium (Thermo Fisher Scientific, Waltham, MA, USA) with 10% fetal bovine serum (HyClone, Logan, UT, USA). All cells were kept in an incubator containing 5% CO_2_ at 37 °C. 

### 4.2. Mouse Models

*Npr1^+/−^* mice were established as previously described (Bioraylab, Shanghai, China) [50]. Genotypes were determined by PCR for toe clipping. An atherosclerosis mouse model was generated by placing *Apoe* knockout (*Apoe^−/−^*) mice (8 weeks) (Vital River, Beijng, China) on a high-fat diet for 9 weeks. Wild-type (WT) mice were fed with a high-fat diet as controls. This research recruited male mice only. 

### 4.3. Total RNA Extraction

Cultured cells or mouse tissues were incubated with Trizol reagent (Bmassay, Beijing, China) and centrifuged at 13,000× *g* for 5 min at 4 °C. The supernatant was collected and mixed with chloroform. After centrifugation, RNA in the aqueous phase was precipitated by mixing with isopropanol at −20 °C for 1 h. Then, the RNA pellet was washed twice and resuspended. The sample was centrifuged at 13,000× *g* for 5 min at 4 °C. The dried pellet was dissolved by DEPC-treated water. The quality and quantity of total RNA were assessed using Nano Drop (Thermo Fisher Scientific, Waltham, MA, USA) and Agilent 2100 Bioanalyzer (Thermo Fisher Scientific, Waltham, MA, USA).

### 4.4. Real-Time Quantitative PCR (qPCR)

Total RNA was reverse transcribed into cDNA using a reverse transcriptase kit (Zomanbio, Beijing, China). qPCR was conducted with M5 HiPer Realtime PCR Super mix (Mei5bio, Beijing, China) using qTOWER3 G Real Time PCR Systems (Analytik Jena, Jena, Germany). The primer sequences used in the study are listed below. Mus-Npr1: forward-5′-AGA CGA TGG GCA GGA TAG G-3′, reverse-5′-GGA AGG ATG CTG GGA TGA T-3′; Mus-Itgb4: forward-5′-GTC GCC GTC TGG TAA ACA T-3′, reverse-5′-ACC TGG TCT CCA CGA CTC AC-3′; Mus-Actin: forward-5′-AGA GGG AAA TCG TGC GTG AC-3′, reverse-5′-CAA GAA GGA AGG CTG GAA AA-3′. The Ct values were obtained according to the amplification curve. The relative expression of Npr1 and Itgb4 was calculated by the 2^−ΔΔCt^ method. 

### 4.5. RNA-Sequencing Analysis

The sequencing data were filtered to produce clean reads using SOAPnuke software. The clean reads were mapped and aligned to the reference coding gene set with Bowtie2 (v2.25). The expression level of genes was evaluated by RSEM (V1.2.12). Differential expression analysis was executed by DESeq2 (v1.4.5) with q value ≤ 0.05. Gene Ontology (GO) and Kyoto Encyclopedia of Genes and Genomes (KEGG) enrichment analyses were carried out by over-representation analysis (ORA) in the WEB-based GEne SeT AnaLysis Toolkit (www.webgestalt.org, accessed on 28 October 2021). 

### 4.6. siRNA Transfection

HUVECs (1.2 × 10^5^) were seeded in six-well plate and cultured overnight. After replacing with Opti-MEM (Thermo Fisher Scientific, Waltham, MA, USA), the cells were transfected with siRNA targeting *NPR1* gene using Lipofectamine 2000 (Thermo Fisher Scientific, Waltham, MA, USA) for 48 h. A non-targeting siRNA was used as a control. The sequences of siRNA were designed and synthesized as follows. Control siRNA: 5′-UUC UCC GAA CGU GUC ACG UTT-3′, si*NPR1*-1: 5′-GCA AAG GCC GAG UUA UCU A-3′, si*NPR1*-2: 5′-CCU AUG AGC AGU UCA ACU U-3′, si*ITGB4*-1: 5′-CCA GGA AGA UCC AUU UCA ATT-3′, si*ITGB4*-2: 5′-GCA CGU GUG AGG AAU GCA ATT-3′. 

### 4.7. Overexpression of NPR1 in Tumor Necrosis Factor-α (TNF-α)-Treated HUVECs

HUVECs were transfected with adenovirus expressing human *NPR1* (ADV-*NPR1*) (Obio, Shanghai, China) for 48 h. Then, the cells were treated with TNF-α at a final concentration of 5 ng/mL (MilliporeSigma, St. Louis, MO, USA) for 24 h and used for additional experiments. 

### 4.8. Immunofluorescent Staining

Frozen sections were fixed with 4% neutral buffered paraformaldehyde (Bmassay, Beijing, China) for 10 min and then blocked with PBST containing 1% BSA for 2 h at room temperature. After blocking, the sections were incubated with primary antibody including NPR1 (1:100, Thermo Fisher Scientific, Waltham, MA, USA), ITGB4 (1:400, Proteintech, Wuhan, Hubei, China), ICAM-1 (1:200, Proteintech, Wuhan, Hubei, China), and CD31 (1:100, R&D, Minneapolis, MN, USA) at 4 °C overnight. Subsequently, the sections were incubated with secondary antibody Cy3-labeled donkey anti-rabbit IgG (1:200, Biolegend. San Diego, CA, USA) or Cy5-affinipure donkey anti-goat IgG (1:200, Jackson ImmunoResearch, West Grove, PA, USA) at room temperature 1 h. The nucleus was stained with Hochest3342 (Beyotime, Shanghai, China). The sections were examined by confocal microscope (Carl Zeiss, Oberkochen, Germany).

### 4.9. Western Blot

Protein from cells was extracted with RIPA buffer (Solarbio, Beijing, China). The sample was loaded onto 12% SDS-PAGE gel for electrophoresis and then transferred onto a PVDF membrane. After blocking by 5% skim milk at room temperature for 2 h, the membrane was incubated with primary antibody targeting NPR1 (1:2000, Thermo Fisher Scientific, Waltham, MA, USA), ITGB4 (1:1000, Proteintech, Wuhan, Hubei, China), ICAM-1 (1:1000, Proteintech, Wuhan, Hubei, China), and GAPDH (1:10,000, Proteintech, Wuhan, Hubei, China) at 4 °C overnight. Afterwards, incubation of secondary antibody HRP goat anti-rabbit IgG (1:10,000, Proteintech, Wuhan, Hubei, China) or HRP goat anti-mouse IgG (1:10,000, Proteintech, Wuhan, Hubei, China) was carried out at room temperature for 2 h. The protein band was detected by Tanon 5200 Chemiluminscent and Fluorescent Imaging System (Tanon, Shanghai, China). 

### 4.10. Monocyte-Endothelial Cell Adhesion Assay

Monocytes (THP-1) were incubated with 10 μmol/L BCECF-AM (Beyotime, Shanghai, China) for 1 h in an incubator with 5% CO_2_ at 37 °C. Then, monocytes were seeded onto HUVECs at a density of 5.0 × 10^6^ cells/mL per well in a 24-well plate. After incubation at 37 °C for 1 h, the non-adherent cells were rinsed off by PBS. The monocyte adhesion to HUVECs was detected by fluorescence microscope (Carl Zeiss, Oberkochen, Germany). 

### 4.11. Oil Red O Staining

The mouse aorta frozen section was fixed with 4% neutral buffered paraformaldehyde (Bmassay, Beijing, China) for 10 min and then rinsed with 60% isopropanol. The section was stained with Oil Red O solution (Beyotime, Shanghai, China) for 15 min. The nucleus was stained with Gill’s hematoxylin (Beyotime, Shanghai, China) for 30 sec. After rinsing with distilled water, the section was mounted in glycerine jelly (Bmassay, Beijing, China).

### 4.12. Measurement of cGMP Production

cGMP concentrations were measured using the Cyclic GMP XP^®^ Assay Kit (CST, Danvers, MA, USA). HUVECs were lysed with 100 µL of lysis buffer. The samples (50 µL) were incubated with 50 µL of cGMP-HRP conjugate for 3 h at room temperature. TMB substrate was then added to the samples for 30 min incubation. After adding the stop solution, the optical density values were determined at 450 nm using SpectraMax i3x (Molecular Devices, San Jose, CA, USA).

### 4.13. Statistical Analysis

All data are presented as the mean ± SD. Statistical analysis was carried out using GraphPad PRISM version 8.4.2 (La Jolla, CA, USA). One-way ANOVA with Dunnett’s multiple comparison was used for three groups and two-tailed Student’s *t*-test was used for two groups. A *p* value < 0.05 indicates statistical significance.

## Data Availability

The RNAseq dataset in this study is available at NCBI’s Gene Expression Omnibus with accession number of GSE215215.

## Abbreviations

NPR1	natriuretic peptide receptor 1
ICAM-1	intercellular adhesion molecule 1
ITGB4	integrin beta 4
ANP	atrial natriuretic peptide
cGMP	cyclic guanosine monophosphate
TNF-α	tumor necrosis factor-α
Tnr	tenascin R
Parvg	parvin gamma
Col6a6	collagen type VI alpha 6 chain
Col6a5	collagen type VI alpha 5 chain
Thbs4	thrombospondin 4
Vtn	vitronectin
